# Female Media Use Behavior and Agreement with Publicly Promoted Agenda-Specific Health Messages

**DOI:** 10.3390/ijerph111212532

**Published:** 2014-12-02

**Authors:** Shu-Yu Lyu, Ruey-Yu Chen, Liang-Ting Tsai, Shih-fan Steve Wang, Feng-En Lo, Ying-Chen Chi, Donald E. Morisky

**Affiliations:** 1 School of Public Health, Taipei Medical University, Taipei 11031, Taiwan; E-Mails: sylyu@tmu.edu.tw (S.-Y.L.); rueyyu@tmu.edu.tw (R.-Y.C.); 2 Graduate Institute of Educational Information and Measurement, National Taichung University of Education, Taichung 40306, Taiwan; E-Mail: liangting.tsai@gmail.com; 3 Department of Journalism, College of Communication, National Chengchi University, Taipei 11605, Taiwan; E-Mail: sfwang@mail.fgu.edu.tw; 4 Department of Leisure and Recreation Management, Asia University, Taichung 41354, Taiwan; E-Mail: lo.fengen@msa.hinet.net; 5 Department of Education & Research, Taipei City Hospital, Taipei 10341, Taiwan; 6 Department of Community Health Sciences, Fielding School of Public Health, University of California at Los Angeles, Los Angeles, CA 90095, USA; E-Mail: dmorisky@ucla.edu

**Keywords:** health information seeking, women, media use, self-efficacy

## Abstract

This study set out to explore the relationship between female media use behavior and agreement with agenda-specific publicly promoted health messages. A random digit dial telephone cross-sectional survey was conducted using a nationally representative sample of female residents aged 25 and over. Respondents’ agreement with health messages was measured by a six-item Health Information Scale (HIS). Data were analyzed using chi-square tests and multiple logistic regression. This survey achieved a response rate of 86% (*n* = 1074). In this study the longest duration of daily television news watching (OR = 2.32), high self-efficacy (OR = 1.56), and greater attention to medical and health news (OR = 5.41) were all correlates of greater agreement with the selected health messages. Surprisingly, Internet use was not significant in the final model. Many women that public health interventions need to be targeting are not receptive to health information that can be accessed through Internet searches. However, they may be more readily targeted by television campaigns. Agenda-specific public health campaigns aiming to empower women to serve as nodes of information transmission and achieve efficient trickle down through the family unit might do better to invest more heavily in television promotion.

## 1. Introduction

The media is a powerful tool for informing the public about health and medical knowledge [1]. Historically, television and other traditional media sources have played major roles in the dissemination of health information [2,3]. However, research over the past decade has indicated a dramatic rise in health information seeking over the Internet, suggesting that it may be subsuming much of the health promotion roles previously held by other forms of media [4,5,6].

While the Internet is a useful tool for some information searches, there is growing concern over the utility of its role in providing sound health information [7]. Access to the Internet is inequitable, and the search for information among those who do have access is hindered by navigational challenges arising from poor organization, technical language, and lack of permanence [7]. Moreover, health information searches on the Internet require that the information seeker makes decisions regarding the utility and reliability of information of questionable quality [8]. Thus, it is also understandable how information seekers lacking basic media literacy skills may easily feel overwhelmed and have difficulty identifying and evaluating the barrage of information presented by even the most specific of Internet searches [9].

This may also be the case among women, who are the most frequent Internet health information seekers [10] and often play important roles as sources of health information in their families, making consumer decisions regarding dietary, lifestyle, and treatment choices [4,11]. Women are often the most common caregiver [12], serving as a surrogate decision-maker, care manager, hands-on health provider, and advocate for their family and friends [13].

This is particularly salient among Taiwanese women who serve as caregivers of their own household and of their husband. However, as these women bear the burden of educating children, caring for an extended family, and maintaining a career, it is difficult to say how to best reach this demographic. Thus, from a public health standpoint, it is important to determine what sources of information can be most effectively used as a platform to reach and inform this demographic of important health concepts to best utilize this health information node and increase information dissemination efficacy. Therefore, in order to enhance agenda-sensitive strategy in health promotion, we conducted this study to explore female media use behavior and its association with their agreement with publicly promoted health messages.

## 2. Methods

### 2.1. Sample and Data Collection

A random digit dial telephone cross-sectional survey was conducted on weekday evenings, from 6:10 P.M. to 10:00 P.M., Monday to Friday, using a nationally representative sample among female community residents aged 25 and over. Data were collected using a computer-assisted telephone interview system by stratified random sampling according to geographic area. The first eligible respondent to answer the phone was interviewed and numbers were considered invalid after three attempts at contact with no response. Of the 8482 dialled calls, 1245 eligible respondents picked up, with 1074 respondents completing the interview. This resulted in a response rate of 86%. The total pool of respondents were weighted according to Taiwanese census data on age and population structure to ensure representativeness. The maximum deviation of sampling error at the 95% confidence level was less than ±3%.

### 2.2. Measures

The survey was implemented using a structured questionnaire. This questionnaire was evaluated by experts in health education, epidemiology, public health, sports medicine, community medicine, and communication. The measurements used for analysis in this study are described below.

#### 2.2.1. Demographic Information

Respondents’ demographic characteristics included age, level of education, occupation, current residence, and average monthly household income.

#### 2.2.2. Media Use Behavior Regarding Medical and Health News 

Media use behavior consisted of the source of health information (television, newspaper, magazine, Internet, radio), frequency of exposure to that information source, and the average length time exposed to that information source. The frequency of attention to medical and health news was measured by the question: “In general, how often do you make an effort to pay attention to medical and health news?” The answer options included “Always”, “often”, “A little“, and “Not at all”. Respondents were also asked to indicate the source of the medical and health news that they received over the one month preceding administration of the interview. Respondents were asked to indicate the average length of time they spent watching television news daily.

#### 2.2.3. Health Information Seeking 

Health information seeking was measured by the question: “In the past month, have you searched for any medical or health information?” Among those who had sought health information, two additional questions were asked to measure how often they provided health information to their family and their level of self-efficacy in information seeking.

#### 2.2.4. Perception of Health Information 

Respondents’ perception of health information was measured by six statements which were selected from the health messages promoted by the Taiwanese Health Department. The selected statements included that, “Women over 30 years old should have a PAP smear annually”, “Second hand smoking is more hazardous than first hand smoking”, “Three servings of vegetables and two servings of fruit per day is helpful for cancer prevention”, “Menopausal women are more prone to having osteoporosis”, “Large-waisted women are at a greater risk of developing cardiovascular diseases and diabetes than women who have large thighs or hips”, and “Three 10-min periods of exercise and one 30-min period of exercise have almost the same health benefit”. These six items were used to construct the Health Information Scale (HIS). Response options included a 5-point Likert scale ranging from “strongly agree” to “strongly disagree”. Subjects who responded that they “Strongly agree” or “Agree” with a given statement received 1 or 2 points, respectively, while subjects who responded that they “disagree” or “strongly disagreed” with a government sponsored health promotion message did not receive any points at all. Those answering “don’t know” were collapsed into the category of “disagree” with a score of 0.

### 2.3. Statistical Analyses

A reliability test was employed to assess the internal consistency of the HIS. A total HIS score of 9 was used as a cut-off point to divide the respondents into two groups, agreement *vs.* non-agreement with publicly promoted health messages. We chose a cut-off point of 9 for the HIS in accordance with other studies, such as those utilizing the Center for Epidemiologic Studies Depression Scale (CES-D), which divided subjects into two groups so that roughly 80% were treated as “functional” and 20% are treated as “dysfunctional” [14]. In this study, while we did not compare disease states, as with the prior study the larger group was used as a reference group with the smaller group being used as the experimental group. This methodology is supported by Rogers’ Diffusion of Innovation Theory which posited that roughly 16% of a given population would be represented by traditional and conservative “laggards” who would most strongly resist new ideas [15]. Frequencies and percentages were applied to the descriptive statistics of the survey data. The chi-square test was used to detect differences between agreements *vs.* non-agreements with publicly promoted health messages. Multiple logistic regression was employed to examine the association between the respondents’ characteristics and HIS. Those variables which were significant in the chi-square analyses were considered significant and included in the multivariate analysis. However, those variables which were highly correlated with one another were not included in the final model as to avoid issues arising due to multicollinearity. We also included age and level of education as control variables in the multivariate analysis. Statistical analyses were performed using SPSS 18.0 statistics software.

## 3. Results and Discussion

### 3.1. Results

A total of 1074 respondents were recruited in this survey, with a response rate of 86%. Roughly 24.3% of the respondents were 25 to 34 years old and 45.7% of the respondents had achieved a high school level education. The majority of respondents (52.6%) were housewives, retired, nor did not have a job, 43.0% of the respondents lived in northern Taiwan, and 42.1% of the respondents had monthly incomes ranging between 30,000 and 59,999 New Taiwan Dollars.

The majority of respondents reported watching 31–60 min of television a day (31.2%), being attentive of medical and health news often (45.2%), seldom providing health information to family members (46.3%), and not seeking health information (42.1%, Table 1).

**Table 1:** Bivariate analysis of respondents’ agreement with publically promoted health messages (N = 1074).

Variable	HIS Score			*p* Value
	>9	≤9	Total	
Age				0.130
25–34	24.2	24.5	24.3	
35–44	22.4	25.9	23.4	
45–54	22.1	25.2	22.9	
55–64	14.5	13.8	14.3	
65 and above	16.7	10.7	15.1	
Level of education				0.002
Primary school or under	22.4	13.9	20.1	
high school	45.9	44.9	45.7	
college and above	31.7	41.1	34.2	
Working Status				0.064
Housewives/Retired/no job	54.1	48.6	52.6	
Working women	45.9	51.4	47.4	
Residential counties/cities				0.548
Northern	42.0	45.7	43.0	
Middle	24.5	23.0	24.1	
Southern	28.0	27.5	27.8	
Eastern	5.5	3.8	5.0	
Average household monthly income (NT$)				0.016
<30,000	22.2	15.4	20.2	
30,000–59,999	42.8	40.6	42.1	
60,000 and above	35.0	44.0	3.7	
Daily television news watching time				0.007
None	7.9	2.8	6.5	
<30 min	21.0	18.0	20.2	
31–60 min	31.8	29.9	31.2	
61–120 min	23.3	28.5	24.8	
>120 min	16.0	20.8	17.3	
Attentive of medical and health news				<0.001
Always	24.6	45.7	30.4	
Most of the time	46.7	41.2	45.2	
A little	17.6	10.7	15.7	
Not at all	11.1	2.4	8.7	
Source of medical and health news				<0.001
Internet	18.4	27.8	20.9	
Television	19.3	27.8	21.6	
Newspapers	5.7	7.2	6.1	
Physician	1.5	3.1	2.0	
Others	51.6	33.0	46.6	
Frequency of providing health information to family members				0.001
Always	8.3	9.6	8.7	
Sometimes	29.4	43.3	34.0	
Seldom	49.3	40.0	46.3	
Never	13.0	6.7	11.0	
Self-efficacy in seeking health information				<0.001
High	33.5	54.3	39.1	
Low	19.3	17.5	18.8	
Did not search	47.2	28.2	42.1	

Percentage was the valid percentage after deleting missing data.

Table 2 presents the distribution of agreement with the publicly promoted health statements. Among the respondents, 67.7% strongly agreed that, “Women over 30 years old should have a PAP smear annually”, and 66.6% strongly agreed that, “Second hand smoke is more hazardous than first hand smoke”. More than half of the respondents strongly agreed with the statement that “Three servings of vegetables and two servings of fruit per day is helpful for cancer prevention” and the statement that, “Menopausal women are more prone to having osteoporosis”.

The lowest mean scores among the six items were with regard to the statements that, “Large-waisted women are at a greater risk of developing cardiovascular diseases and diabetes than women who have large thighs or hips” and, “Three 10-min periods of exercise and one 30-min period of exercise have almost the same health benefit”.

Table 3 illustrates the associations between various respondent characteristics and an HIS of above 9, corresonding to approximately the upper 20% of the score distribtuion. Multiple logistic regression analysis revealed that subjects with high self-efficacy (OR = 1.56, 95%CI = 1.09‒2.23), a daily television news habit of watching over120 min (OR = 2.32, 95%CI = 1.01‒5.29), and those who reported always or often being attentive of medical and health news (OR = 5.41, 95%CI = 2.13‒13.76, OR = 2.84, 95%CI = 1.14‒7.07) were more likely to fall in the upper 20% of the HIS distribution after controlling for respondents’ age, working status and level of education.

**Table 2:** Distribution of agreement with publically promoted health messages ^a^ (*N* = 1074).

Item	Strongly Agree N (%)	Agree N (%)	Disagree/Strongly Disagree ^b^ N (%)	Mean Score Mean ± SD
Women over 30 years old should have a PAP smear annually	727(67.69)	247(23.00)	100(9.31)	1.58 ± 0.66
Second hand smoke is more hazardous than first hand smoke	715(66.57)	228(21.23)	131(12.20)	1.54 ± 0.70
Three servings of vegetables and two servings of fruit per day is helpful for cancer prevention	577(53.72)	374(34.82)	123(11.46)	1.42 ± 0.69
Menopausal women are more prone to having osteoporosis	573(53.35)	311(28.96)	190(17.69)	1.36 ± 0.76
Large-waisted women are at a greater risk of developing cardiovascular diseases and diabetes than women who have large thighs or hips	365(33.99)	341(31.75)	368(34.26)	1.00 ± 0.83
Three 10-min periods of exercise and one 30-min period of exercise have almost the same health benefit	157(14.62)	338(31.47)	578(53.91)	0.61 ± 0.73
Total				7.51 ± 2.73

^a^ Cronbach’s alpha = 0.687; ^b^ including “Don’t know”.

**Table 3:** Association between respondent characteristics and a high Health Information Score (reference group: HIS score ≤ 9) (*N* = 1074).

Variable	OR (95.0% CI)	*p*
Age		
25–34	1.06(0.71‒1.59)	0.778
35–44	1.05(0.69‒1.60)	0.800
45–54	1.16(0.67‒1.99)	0.605
55–64	1.14(0.61‒2.12)	0.681
65 and above	1	
Level of education		
Primary school or under	0.92(0.53‒1.61)	0.769
high school	0.94(0.68‒1.30)	0.700
college and above	1	
Self-efficacy		
High ^*^	1.56(1.09‒2.23)	0.016
Low	1.05(0.68‒1.61)	0.830
Did not search	1	
Daily television news watching time		
>120 min ^*^	2.32(1.01‒5.29)	0.046
61–120 min	2.09(0.94‒4.68)	0.072
31–60 min	1.69(0.76‒3.74)	0.198
<30 min	1.74(0.77‒3.96)	0.184
None	1	
Attentive of medical and health news		
Always ^***^	5.41(2.13‒13.76)	<0.001
Most of the time ^*^	2.84(1.14‒7.07)	0.025
A little	2.21(0.85‒5.77)	0.106
Not at all	1	

^*^ *p* < 0.05; ^***^ *p* < 0.001.

### 3.2. Discussion

This study set out to explore the relationship between female media use behavior and agreement with agenda-specific health messages promoted by the Taiwanese Health Department. We found the duration of daily television news watching, self-efficacy, and greater attention to medical and health news to all be correlates of greater agreement with publicly promoted health messages. Surprisingly, Internet use was not significant in the final model.

In this study we specifically investigated the associations between media use and agreement with publicly promoted health messages to better explore how to best inform the public of agenda-specific issues. Women were chosen as the target population as they have a central role in making health and consumer decisions, as well as managing the health of their families [16], and it is therefore efficient to reach this demographic to better disseminate information to the overall population through the trickle-down effect targeting the family unit. This is particularly the case among Taiwanese women who are largely responsible for the care and consumer decisions of both their own household as well as that of their husband [17], leaving them a particularly important node of information transmission to consider. However, on account of having to balance the responsibility of educating children and caring for an extended family in addition to maintaining a career [16], it is difficult to say how to best reach this demographic.

The results of this study suggest that many women which public health interventions need to be targeting are not receptive to health information that can be accessed through Internet searches, and to make use of women as a node of information transmission and achieve efficient trickle down through the family unit, agenda-specific public health campaigns might do better to invest more heavily in television than internet promotion. These conclusions are in line with those of a recent study conducted in the United States which underscored the continued importance of traditional media, despite the Internet revolution [18].

There are several potential reasons which may underlie the association detected between the duration of television watching and the greater agreement with public health messages, as well as our inability to detect an association between Internet use and the same outcome. The first regards the dynamic surrounding the nature of the information contained in the public health messages, the interest of our population to seek out this information actively, and the specificity of the information received through these two mediums. The second is the greater access to television information than Internet information, and the third, which is related to the second, is the level of media literacy required to effectively sort through the results of an Internet search.

While Internet searches can be a valuable asset toward ascertaining specific answers to specific health queries, their utility only becomes apparent when an individual actively seeks out information. This underscores a major obstacle to the efficient dissemination of public health information using the Internet: Public health messages can only reach an audience who is actively seeking out the promoted information. As most people may not be vigilant of public health threats [19], and only casually read through recently published public health announcements at best, Internet campaigns may not be the most effective method to conduct health promotion or health education. In fact, the very people that public health interventions often seek to target are also those who may not be likely, or may not be able, to actively seek out health information [20].

The second reason that television but not Internet use may have been correlated with a greater agreement with publicly promoted public health messages may involve the problem of access. There is certainly inequality in access to the Internet, which requires both hardware and an Internet source [8].These prerequisites to Internet access leave lower socioeconomic class individuals at an information disadvantage. This is a particular problem for public health professionals battling against the “inverse care law” which posits that socio-economic inequalities in health may arise from differential uptake of preventive and therapeutic medical services, leaving the lower socioeconomic classes an important target for public health campaigns [21]. While in this study, the subjects who did not use the Internet, and therefore most likely did not have access to the Internet, were not included in the analysis of the correlation between Internet use and agreement, access to the Internet does not indicate that an information seeker is able to access the appropriate information. It is possible that the difficulty of navigating the Internet may preclude an Internet user from accessing it. But, even if successfully navigated, deciding which information is correct and accessing the knowledge it contains requires interpretation and media literacy [22].

The third reason that Internet use was not correlated with a greater agreement with publicly promoted public health messages while television news viewing time was, may have been that a lower level of media literacy was required to view and interpret the health messages presented by television news. As the media in general is subject to biased reporting of health issues, and the Internet presents a plethora of often contrasting and disorganized information from varying sources, it is possible that the more singular, authoritative, and “boiled down” public health message presented by television news might have been far more digestible for the women in this study [23].

Therefore, the women who in this study who were avid Internet users may have largely been able to navigate to their desired websites with a minimum of interruptions, thus skillfully avoiding opportunities to be confronted by public health messages. On the other hand, the women who were less than avid users of the Internet were likely to have been unable to locate credible information, and may have either been unable to actively use the Internet to search for information, or sought out incorrect information on account of lacking the media literacy and technological savvy to know the difference. The final category of women, those who did not have any access to the Internet, would be completely naïve to any Internet-based campaign.

In contrast to the Internet, access to television is far more prevalent. Moreover, television watchers have far less control over what they watch, and are victim to pre-scheduled programming. Thus, anyone watching a news program, whether or not they are looking for health information, will still have the benefit of being exposed to public health announcements if broadcast during the duration of the program. However, this study did also detect that subjects who were actively attentive of medical and health news were more likely to have greater agreement with publically promoted health messages.

In this study we also found that women with greater self-efficacy were more likely to have greater agreement with the publically promoted health messages than those women reporting lower levels of self-efficacy. These findings are in agreement with previous studies which found self-efficacy to be associated with health literacy [24,25]. This may go to demonstrate that simply providing the pertinent information to women may not be sufficient, and that a public health campaign targeting a particular objective among the female demographic may need to further empower women. This adds further evidence that more traditional health communication techniques which often intend to persuade or nudge women may not be as efficient as other methods that are more informative, and utilize nondirective approaches which aim to empower through education [26].

While this study succeeded in detecting associations between daily television news watching, self-efficacy, and a greater attention to medical and health news with a greater agreement with publically promoted health messages, these results need to be interpreted in the light of several limitations. The first limitation involves this study’s use of cross-sectional data which limited our ability to make causal inferences. Secondly, this study utilized a random digit dial telephone survey, and Taiwanese who did not have a telephone at the time this study was condcuted would have not have been included in our analysis. While this may have contributed to a selection bias, technological penetration is high in Taiwan, and apart from a small proportion of the population of indigenous peoples living on tribal lands (<1%) nearly everyone has a telephone. Finally, as phone numbers were considered invalid after three attempts were made without contacting an eligible subject, its possible that we oversampled housewives. However, this study achieved a response rate of 86%, so it is unlikely that this seriously affected the results of this study.

## 4. Conclusions

This study reported that the duration of daily television news watching, self-efficacy, and greater attention to medical and health news were all correlates of greater agreement with publically promoted health messages, while Internet use was not. The results of this study have implications for health marketing strategies using media channels among female residents and future research should address these hypotheses in greater detail and further investigate how media and Internet literacy influences receptiveness to agenda-specific public health messages among women.

## Practice Implication

Agenda-specific public health campaigns aiming to make use of women as a node of information transmission and achieve efficient trickle down through the family unit might do better to invest more heavily in television promotion.
